# AnGeLi: A Tool for the Analysis of Gene Lists from Fission Yeast

**DOI:** 10.3389/fgene.2015.00330

**Published:** 2015-11-16

**Authors:** Danny A. Bitton, Falk Schubert, Shoumit Dey, Michal Okoniewski, Graeme C. Smith, Sanjay Khadayate, Vera Pancaldi, Valerie Wood, Jürg Bähler

**Affiliations:** ^1^Research Department of Genetics, Evolution and Environment – UCL Genetics Institute, University College LondonLondon, UK; ^2^Scientific IT Services, ETH ZürichZürich, Switzerland; ^3^Cambridge Systems Biology and Department of Biochemistry, University of CambridgeCambridge, UK

**Keywords:** gene cluster, ontology, *S. pombe*, PomBase, data mining, database, large-scale assay, genetic screen

## Abstract

Genome-wide assays and screens typically result in large lists of genes or proteins. Enrichments of functional or other biological properties within such lists can provide valuable insights and testable hypotheses. To systematically detect these enrichments can be challenging and time-consuming, because relevant data to compare against query gene lists are spread over many different sources. We have developed AnGeLi (Analysis of Gene Lists), an intuitive, integrated web-tool for comprehensive and customized interrogation of gene lists from the fission yeast, *Schizosaccharomyces pombe*. AnGeLi searches for significant enrichments among multiple qualitative and quantitative information sources, including gene and phenotype ontologies, genetic and protein interactions, numerous features of genes, transcripts, translation, and proteins such as copy numbers, chromosomal positions, genetic diversity, RNA polymerase II and ribosome occupancy, localization, conservation, half-lives, domains, and molecular weight among others, as well as diverse sets of genes that are co-regulated or lead to the same phenotypes when mutated. AnGeLi uses robust statistics which can be tailored to specific needs. It also provides the option to upload user-defined gene sets to compare against the query list. Through an integrated data submission form, AnGeLi encourages the community to contribute additional curated gene lists to further increase the usefulness of this resource and to get the most from the ever increasing large-scale experiments. AnGeLi offers a rigorous yet flexible statistical analysis platform for rich insights into functional enrichments and biological context for query gene lists, thus providing a powerful exploratory tool through which *S. pombe* researchers can uncover fresh perspectives and unexpected connections from genomic data. AnGeLi is freely available at: www.bahlerlab.info/AnGeLi

## Introduction

Large-scale and genome-wide studies such as the profiling of gene expression, DNA-binding sites, mutant phenotypes, or genetic interactions, typically lead to sizeable lists of candidate genes or proteins. Such gene lists often contain valuable, hidden biological information which can enlighten the processes studied, provide useful context, and generate testable hypotheses for targeted follow-up experiments. While the generation of gene lists entails established experimental and analytical procedures, the extraction of any biologically meaningful information from such lists can be a serious challenge. Evidently, prior knowledge is a major factor affecting interpretation of any gene list, regardless of the underlying biological or experimental context by which it was generated. Gene list interpretation therefore relies on the availability of comprehensive reference information on genes and proteins against which the list can be compared to uncover any statistically significant common features among its members.

To get the most from gene lists, such reference information may include validated or predicted gene/protein functions, detailed data on gene architecture and conservation, regulatory factors, expression levels and context, cellular localization, pathway information, physical/genetic interactions, and phenotypic data, to name just a few. Such databases of integrated gene and protein information are partially provided through InterMine for some organisms but not for fission yeast (Kalderimis et al., 2014). Hence, gene list interpretation relies on incomplete functional annotation databases, combined with statistical tools, which typically interrogate one or more properties in search for any significant enrichment. GO enrichment tools are popular (Ashburner et al., 2000; Boyle et al., 2004; Carbon et al., 2009), which look for over-representation of associated GO terms within gene lists. To meet the growing needs of biologists in the omics era, more specialized gene identifier-based search engines have been developed for various model organisms, including free or commercial resources such as functional annotation tools (Huang da et al., 2009), pathway mapping algorithms (Kanehisa and Goto, 2000; Nikolsky and Bryant, 2009; Kelder et al., 2012; Mi et al., 2013; Croft et al., 2014), or protein interaction search tools (Stark et al., 2006).

The emergence of central, regularly maintained and updated databases that store genomic variation, ontology, pathway, interaction or phenotypic data has attracted software developers to design ‘all-in-one’ search engines that enable systematic searches against published, pre-defined gene sets (Subramanian et al., 2005) and/or multiple functional annotation resources (Zhang et al., 2005; Araki et al., 2012). Such gene set enrichment tools have proven valuable for downstream analysis of large-scale experiments by providing functional insights for query gene lists of interest. Given the rapid growth of relevant information, integration of developer-curated and user-defined gene sets into a single resource offers a flexible solution. GSEA (Subramanian et al., 2005), for example, a standalone or web-based application for selected vertebrates, allows the user to search a query gene list against thousands of curated gene sets but also against additional user-defined lists.

The fission yeast *Schizosaccharomyces pombe* is an important model organism that shares many critical biological processes with multicellular eukaryotes (Wood et al., 2002). Over the years, the fission yeast community has produced many genomic data sets and resources, including a gene deletion collection (Kim et al., 2010; Chen et al., 2015) and protein localization data (Matsuyama et al., 2006). The curators at PomBase [the *S. pombe* model organism database (Wood et al., 2012; McDowall et al., 2015)], are assembling rich information on gene characteristics and functions and on mutant phenotypes by applying the FYPO (Harris et al., 2013). These efforts are supported by volunteer expert curators among the fission yeast community, using the Canto online tool (Rutherford et al., 2014).

We have exploited the rich published and annotated resources to build a generic gene list enrichment tool, AnGeLi, that can satisfy the growing need of the community for a comprehensive, one-stop analysis of gene lists. AnGeLi is an intuitive web-based tool which offers customized analyses of gene lists, by systematically screening a multitude of data sources, including published and user-defined gene sets to highlight statistically significant enrichments. Moreover, AnGeLi encourages a community-wide effort to further increase its usefulness by contributing additional published or otherwise annotated gene lists via its data submission feature. The more data are included in AnGeLi the more powerful it will become in uncovering functional insights, context and unexpected connections, and thus fully unleashing the information hidden in genomic data that currently remain only partially explored.

## Overview of AnGeLi Tool

### Database, Data Types, and Gene Set Resources

AnGeLi is a knowledge-driven, web-based application implemented in Perl. It takes as an input a list of systematic gene identifiers and searches for any enrichment of common features using a diverse collection of annotated resources, published gene lists, or curated gene sets or features (henceforth AnGeLi’s database), as well as user-defined gene sets (optional). AnGeLi’s database includes three discrete types of data: categorical, metric, and pairwise (**Table 1**). Categorical data refer to gene sets representing membership in specific biological categories, where gene membership of a category is stored in binary format. These gene sets are derived from different sources such as specific GO categories, phenotypes, or published gene lists, as examples. A query gene can either belong to a specific gene set (gene value is 1) or not (gene value is 0). Metric data describe a quantifiable, continuous characteristic of a gene or protein such as intron number, distance from centromere or transcript copy number, to name a few examples. Both categorical and metric data are organized in a tabular format prior to data compilation (**Table 1**). Pairwise data represent pair relationships such as genetic or protein–protein interactions.

**Table 1:** Organization of data types (binary and metric) and grouping into themes.

Feature ID	GO:0007126	FYPO:0002061	mRNA-cpc	pI	…
Feature name	Meiotic nuclear division	Inviable vegetative cell population	mRNA copies per-cell	Isoelectric point	…
Feature group	GO Biological Process	Phenotypes (FYPO)	Transcript features	Protein features	…
Data type	Categorical/Binary	Categorical/Binary	Metric	Metric	…
Source	GO Biological Process	Phenotypes (FYPO)	Marguerat et al., 2012	PomBase	…
Gene 1	0	1	0.1	9.9	…
Gene 2	0	0	0.041	5.4	…
Gene 3	0	1	0.47	6.7	…
Gene 4	1	0	0.34	7.7	…
…	…	…	…	…	…

*GO, Gene Ontology; FYPO, Fission Yeast Phenotype Ontology; cpc, copies per cell; pI, isoelectric point.*

AnGeLi’s database currently holds 9632 features (9579 binary, 49 metric, and 4 pairwise features; Supplementary Table S1). These features are sourced directly from PomBase, or calculated using sequence or annotation data (55 features). Other data sources include GO categories (5603 features: 3529 Biological Process; 1277 Molecular Function; 797 Cellular Component; Wood et al., 2012), phenotypes (FYPO; 2682 features; Harris et al., 2013), Pfam domains (1130 features; Finn et al., 2014), and BioGrid interactions (four features; Breitkreutz et al., 2008). To augment AnGeLi’s database beyond the annotated resources, we have initially selected 23 genomic papers which report fundamental expression or functional profiling data (158 features); many more such data can be added in the future using a straightforward submission form (see below). Among the categorical data, we included gene lists from defined ‘housekeeping’ genes (Pancaldi et al., 2010), stress-response genes (Chen et al., 2003, 2008; Tanay et al., 2005), meiotic differentiation genes (Mata et al., 2002, 2007; Tanay et al., 2005; Mata and Bähler, 2006), and cell cycle-regulated genes (Rustici et al., 2004; Marguerat et al., 2006), genes regulated in chromatin mutants (De Groot et al., 2003; Tanny et al., 2007) or in response to caffeine and rapamycin (Rallis et al., 2013), and gene sets that highlight differences between haploid and diploid transcriptomes (Bitton et al., 2011). We also incorporated key regulatory modules (Tanay et al., 2005), transcription factor targets (Rustici et al., 2004; Tanay et al., 2005), protein localization data (Matsuyama et al., 2006), genes identified in genome-wide splicing assays (Bitton et al., 2014, 2015), targets of RNA-binding proteins (Lemieux et al., 2011; Hasan et al., 2014), GPI-anchored cell-surface proteins (De Groot et al., 2003; Tanny et al., 2007), as well as genes involved in TORC1 function, lifespan and growth (Rallis et al., 2014; Sideri et al., 2014). Among the metric data, we incorporated genetic diversity among wild *S. pombe* strains (Jeffares et al., 2015), transcript half-life data (Amorim et al., 2010; Hasan et al., 2014), RNA polymerase II occupancy (Lackner et al., 2007), cellular transcript and protein copy numbers (Marguerat et al., 2012), protein molecular weight, amino acid composition, ribosome occupancy, and density (Lackner et al., 2007), AUG translation initiation index (Miyasaka, 2002; Lackner et al., 2007) poly-A tail lengths (Beilharz and Preiss, 2007; Lackner et al., 2007), protein half-lives (Christiano et al., 2014), as well as protein fold-index (Prilusky et al., 2005), which predicts intrinsically unfolded proteins (Gsponer et al., 2008). AnGeLi also stores interaction data from BioGrid (Breitkreutz et al., 2008), including protein–protein and genetic interactions identified in fission yeast, and inferred interactions based on orthologs in budding yeast (Wood, 2006). AnGeLi may thus facilitate the discovery of protein complexes, network ‘hubs’, or enrichment of specific pathways among the query genes.

AnGeLi’s output is grouped in themes capturing different biological aspects: GO categories, Gene Expression (differentially regulated genes under different conditions), Gene Features (e.g., intron number, chromosomal position, and genetic diversity), Genetic and Physical Interactions (based on BioGRID), Phenotypes (based on FYPO), Phenotypic Profiles (genes identified in mutant screens), Protein Domains (based on Pfam), Protein Features (e.g., amino-acid composition, conservation, and cellular copy numbers), Protein Localizations (based on ORFeome), and Transcript Features (e.g., RNA length and type, ribosome occupancy, and cellular copy numbers). This grouping into themes facilitates an overview of the results but is not used for any higher-level analysis.

### Statistical Framework for Gene Enrichment Analyses

To determine whether a feature is significantly enriched or under-enriched in the query gene list, AnGeLi automatically selects from three statistical tests depending on the data type. Categorical data are countable (i.e., number of overlapping genes between the query list A and categorical set C), and AnGeLi applies a widely used test for gene set enrichment, the 2-tailed Fisher’s exact test (Rivals et al., 2007). AnGeLi thus determines whether the proportion of genes from set C found in the query list A is significantly higher or lower than the proportion of genes from set C in the entire background gene population. The statistics therefore is affected by the background gene population, which can be adjusted to best match the analysis (see below). Metric data are continuous (e.g., transcript length, copy numbers), and AnGeLi performs a 2-sided Wilcoxon rank-sum test to assess whether the values of metric feature M associated with the genes in query list A are significantly higher or lower than the values of feature M associated with the genes not present in list A. Pairwise data are assessed by a permutation test (Good, 2000) to reveal any enrichments of protein–protein or genetic interactions within the query list. Briefly, a random set of genes (of same gene number as list A) is iteratively drawn from a pool of genes not found in list A and evaluated for protein–protein or genetic interactions in pairwise gene set P; the number of permutations is determined by the user (default is 1000), while the *p*-value is derived from the number of times the random set achieved a greater sum of interactions in set P than the sum of interactions in query list A. Owing to the large increase in analysis time, AnGeLi does not analyze pairwise as a default.

Under default settings, the query list is tested against 7554 features simultaneously (7505 binary and 49 metric features, excluding user-defined gene sets); thus, the probability of false positive enrichments is quite high. To account for this multiple testing problem, AnGeLi provides three approaches for *P*-value correction. The Bonferroni method (Shaffer, 1995) is conservative and simply multiplies the original *p*-value by the total number of tests performed to derive the corrected *p*-value. The Holm (1979) method of correction reduces false negatives, but is still conservative; in brief, the *p*-values are ranked in an ascending order, and the first *p*-value is multiplied by the total number of tests, while each sequential *p*-value is multiplied by a decreasing number of the remaining tests (i.e., *p*-value_1_ x ‘t’ [total number of tests], *p*-value_2_ x [‘t’ – 1], *etc.*). The FDR (Benjamini and Hochberg, 1995) is used as the default option by AnGeLi. FDR is less conservative because it controls the number of false positives in the reported list of significant features. Again, the *p*-values are ranked in ascending order and the corrected *p*-value is equal to the rank divided by the total number of tests performed, multiplied by the accepted false positive threshold chosen by the user. At an FDR of 0.01, we expect 1% false positives among the reported significant features. Note that for the pairwise data type, the *p*-value is highly dependent on the number of permutations set by the user, which in turn dictates the analysis time (see Materials and Methods). When the number of permutations is relatively low (e.g., 1000), even the lowest *p*-value will not be sufficient to pass multiple testing corrections; AnGeLi therefore provides the option to increase the number of permutations at the expense of analysis time. Furthermore, AnGeLi permits deselecting categories that are not of interest, which in turn will increase the statistical power and enhance identification of subtle enrichments, and is therefore recommended if applicable.

**FIGURE 1:**
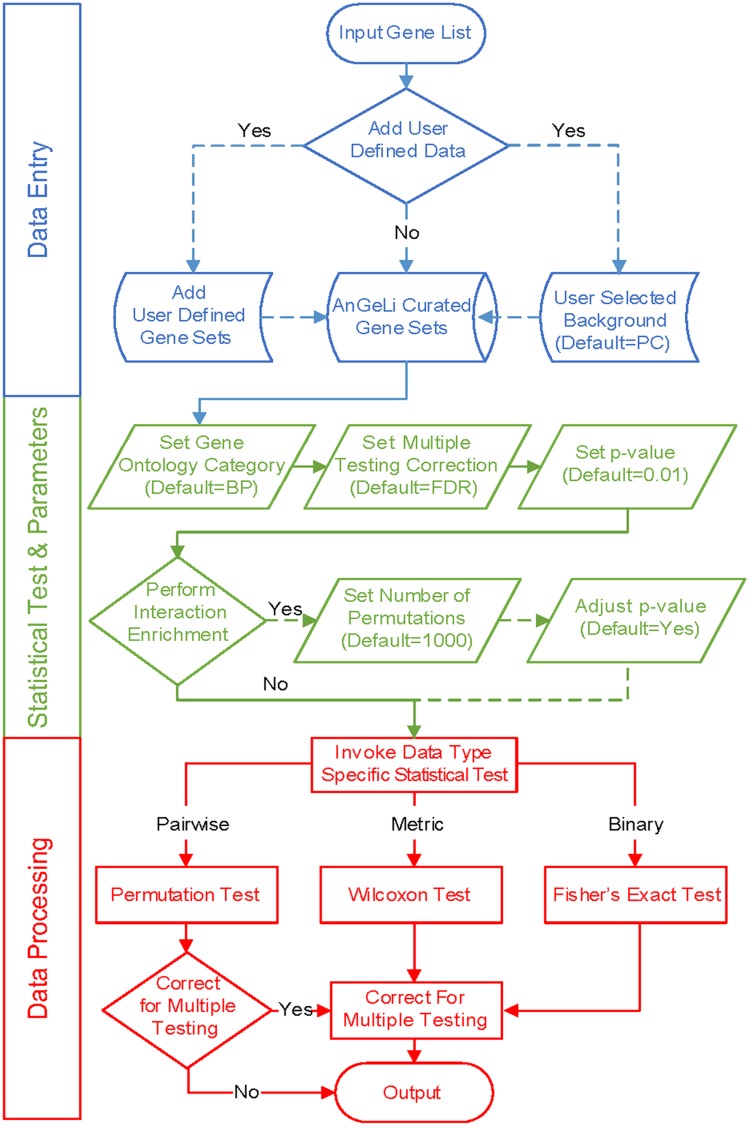
**Workflows in AnGeLi.** (**Top** – blue) Data entry: the user pastes a query gene list and has the option to add user-defined gene sets and/or select the background gene set (default = PC; protein-coding genes). If no additional gene sets are added, under default settings, 7554 features of the AnGeLi knowledgebase will be analyzed (7505 binary, and 49 metric features), because 1277 GO Molecular Function, 797 GO Cellular Component, and 4 Genetic and Physical Interactions (BioGRID) features are excluded by default (9632 features in total). If any user-defined gene sets are added, the database is augmented accordingly. (**Middle** – green) Statistical parameter settings: the user selects GO category (default = BP; Biological Process), a method for multiple testing correction (default = FDR) and the desired *p*-value threshold (default = 0.01). The users can also specify whether to perform the pairwise interaction enrichment analysis (default = No), set the desired number of permutations accordingly (default = 1000), and adjust the *p*-value to account for multiple testing. (**Bottom** – red) Data processing: AnGeLi performs gene list enrichment analysis based on user input and reports any significant functional enriched features, along with associated information.

AnGeLi provides the ability to choose a background gene population as a reference for the statistical analyses based on the query gene list. This option allows tailoring of the analysis to the context of the gene list of interest, which can greatly increase the accuracy and sensitivity of the analysis. For example, a query list from an experiment which has only considered protein-coding genes should be analyzed with the protein-coding gene background. As another example, query genes derived from phenotypic screens with the deletion mutant library will all be non-essential, which would skew the statistics if all genes were used as background. AnGeLi offers six pre-set background options, covering all common scenarios: protein-coding genes (default), all annotated genes, non-coding RNA genes, genes with associated GO terms, genes with associated phenotypes, and non-essential genes. In addition, users can provide their own bespoke background gene list to tailor the analysis to their particular requirements. An overview of AnGeLi’s steps for data entry, statistical tests and data processing is presented in **Figure 1**.

### Comparison to Other Tools and Applications

The breath of AnGeLi offers several advantages over existing tools that are based on only one or two data types such as GO categories or pathways. We compared AnGeLi’s performance to two other tools that support GO enrichment analysis for fission yeast, GeneCodis (Carmona-Saez et al., 2007) and GO Term Finder (Boyle et al., 2004). We assembled a list of 100 protein-coding genes containing 50 cell cycle-regulated genes (Rustici et al., 2004) and 50 random genes (Supplementary Table S2). This list was analyzed with all three tools using FDR as the multiple-testing correction method, with a cutoff of <0.01, using all genes with GO terms as background and Biological Process as category. Surprisingly, GeneCodis did not identify any enrichment in the list, even after disabling the multiple-testing correction option. The GeneCodis database for fission yeast was last updated in December 2011, which could partially explain the lack of any enrichment. On the other hand, the results obtained from AnGeLi and GO Term Finder corresponded very well, with only minor differences (Supplementary Tables S3 and S4): of the 17 enrichments found by AnGeLi, 15 were also found by GO Term Finder which reported numerous additional enrichments with lower significance. These differences between the two tools largely arise from differences in statistical tests and thresholds. AnGeLi actually did find all enrichments presented by GO Term Finder after relaxing the FDR to <0.08.

Importantly, AnGeLi offers a uniquely broad analysis tailored to fission yeast, far beyond GO term enrichments. Enrichments for several informative features are exclusive to AnGeLi, like gene expression signatures and phenotype annotations; the absolute number of phenotype annotations exceeds the number of GO annotations and is currently increasing at a rate of ∼1000 per year. When analyzing the test list of 100 genes with AnGeLi using default settings, rich additional biological insights were provided (Supplementary Table S5). For example, the analysis revealed enrichment in target genes for specific transcription factors that control gene expression during distinct phases of the cell cycle. As another example, the list was associated with abnormal cell-cycle phenotypes, like aberrant mitosis and cell division, and was also enriched for cell surface proteins. AnGeLi has served our group and collaborators very well in numerous studies to obtain biologically meaningful insights from large gene lists. As recent examples, the tool has uncovered helpful functional enrichments, besides GO categories, among lifespan and growth mutants (Sideri et al., 2014) and among the targets of RNA-binding proteins (Cotobal et al., 2015).

AnGeLi provides additional advantages compared to other enrichment tools. It is easily configurable for additional data sets, and users can incorporate their own gene sets. It also provides a broad choice for statistical analyses. Moreover, because of its link with PomBase, users can be assured that AnGeLi remains updated and uses current data. On the other hand, AnGeLi is organism specific and therefore its application is narrower than for other tools, but other organism communities may benefit from similar tools which are configured as a one-stop resource for datasets of specific interest.

### User Interface

AnGeLi offers an intuitive online interface: www.bahlerlab.info/AnGeLi. The user supplies a query gene list (systematic names only), and sets the statistical parameters and background gene list. In addition, users can provide additional gene sets in tab-delimited format. AnGeLi’s output is organized into different themes and include hyperlinks to the corresponding resource or the publication from which the data derive. AnGeLi allows the user to de-select any pre-defined themes; in the extreme case, AnGeLi’s statistical framework could just be used to analyze a query list against a user-defined gene set.

Once analysis is completed, AnGeLi reports a summary of all tests performed, including color-coded tables where over- and under-represented sets and features are highlighted in red and green, respectively. For each theme, enrichments are ranked by their *p*-values, with expected vs. observed gene overlaps provided for categorical data, average values for metric data, and the number of interactions for pairwise data. Only gene sets or features that show any enrichments or under-enrichments are listed. An explain button next to each over- or under-represented gene set or feature provides a detailed summary for the corresponding enrichment. The user can export the results page in tab-delimited format, which also includes the corresponding external database identifiers as well as the actual intersection between the gene sets. A detailed Help Page is also provided (Link *‘Help’*).

To further extend the data types available in AnGeLi, users are encouraged to submit published gene lists through a straightforward submission form. AnGeLi’s database will be updated monthly via synchronization with the annmap annotation package (Yates et al., 2008). The database could be downloaded via a link from the website (Link *‘Download Database’*). Furthermore, user feedback will be monitored via the GitHub issue-tracking utility to allow continuous improvement (Link *’Report a bug’*).

## Materials and Methods

### Database Construction and Resources

AnGeLi utilizes the *S. pombe* Ensembl annotation database (version 27) as the source for gene features (Kersey et al., 2010), which is based on PomBase (McDowall et al., 2015) and is implemented in the annmap core Bioconductor/R package (Gentleman et al., 2004; Yates et al., 2008). The database was used to derive the following: list of genes, exons, proteins, and their chromosomal positions as well as transcript biotypes (i.e., protein-coding, ncRNA, etc.). Applying customized R and Perl scripts, these data were used to compute relative and absolute distances from centromere and telomeres. Similarly, these data were used to compute intron locations, intron number per gene, average intron length and total transcript length. The GC content of the first intron was computed using the ‘geecee’ function within the EMBOSS (Rice et al., 2000). The protein sequence data was downloaded from PomBase (McDowall et al., 2015), and protein features such as molecular weight, isoelectric point, charge, and number of amino acids were also calculated using the EMBOSS suite (pepstats function). Amino acid compositions were calculated using a customized Perl script. The fold-index for each fission yeast protein was computed using a modified Perl script available from http://bip.weizmann.ac.il/fldbin/findex (Prilusky et al., 2005). *S. pombe* GO annotations and the generic GO OBO flat file were downloaded from ftp://ftp.geneontology.org. A recursive algorithm was used to map genes to all corresponding ancestor terms in the ontology. Pfam domains (Finn et al., 2014) were retrieved from the xmapcore database (Yates et al., 2008). For phenotype mappings (Harris et al., 2013), we used the phenotype annotation ‘phaf’ file available from ftp://ftp.ebi.ac.uk/pub/databases/pombase/pombe/Phenotype_annotations/phenotype_annotations.pombase.phaf.gz, fypo OBO file available from https://cdn.rawgit.com/pombase/fypo/master/release/fypo.obo. We only considered GO terms, Pfam domains and phenotypes that were associated with at least two genes. The manually curated human and budding yeast orthologs of fission yeast proteins (Wood, 2006) were retrieved from ftp://ftp.ebi.ac.uk/pub/databases/pombase/pombe/orthologs/cerevisiae-orthologs.txt. Physical and genetic interaction data were downloaded from BioGRID (Breitkreutz et al., 2008) and processed using customized Perl scripts. All binary and metric data were combined into a single table using an R script (similar to **Table 1**) prior to conversion into Perl associative array data structures. Pairwise relationships were stored directly in Perl data structures.

### Implementation of Statistical Tests

All statistical tests and multiple testing correction functions were implemented in Perl. For Fisher’s exact test, the Text::NSP::Measures::2D::Fisher::twotailed module was used (available from http://search.cpan.org), where the 2 × 2 contingency table was constructed using the following values: (row1) genes found in input list ‘A’ and in gene set ‘G’, genes found in gene set ‘G’ but not in input list ‘A’, (row2) genes found in input list ‘A’ but not in gene set ‘G’ and genes not found in input list ‘A’ and not in gene set ‘G’.

The core of the Wilcoxon rank sum test implemented in Perl was adopted from http://www.fon.hum.uva.nl/rob/SignedRank/. In this script, a normal approximation with a continuity correction or an exact test is used, depending on the number of permutations (‘*k*’ out of ‘*n*’) and estimation of the *p*-value. AnGeLi displays a warning for small gene lists (below 10 genes), for permutations ≥2500 or for *p* ≥ 0.25. Genes with no values are ignored throughout.

The pairwise permutation test repeatedly draws a random set of genes from a pool of genes not found in the query list, while the number of permutations is set by the user and the size of the random set is equal to the size of the query list. However, the pool of genes has to be at least twice as large as the query list, otherwise AnGeLi will display a warning that the query list is too large and *p*-values cannot be computed. The running time of the permutation test is quadratic, therefore pairwise analysis is excluded by default and, if selected, permutations are set to 1000. The *p*-value is equal to the number of times the random set has a greater sum of interactions compared to the real set divided by the total number of permutations and multiplied by 2 (i.e., pairwise). For example, in the best-case scenario, where the sum of random interactions equals 0 or 1 following 1000 permutations, the *p*-value will be equal to (1/1000)^∗^2 = 0.002. This relatively high *p*-value is unlikely to be significant following correction for multiple testing (7554 tests: 7505 binary, 49 metric, and 4 pairwise features), and a higher number of permutations at the expense of analysis time should be set.

## Conclusion

AnGeLi offers a unique and flexible statistical framework for the analysis of gene lists derived from *S. pombe*, using a rich catalog of annotated features, published information and gene sets that span multiple and diverse biological aspects. The analyses can be tailored to the query gene lists and enhanced by the addition of user-defined gene sets. With respect to published gene sets, the current content of AnGeLi’s database is somewhat arbitrary and far from complete. We encourage a community-wide effort to further increase the usefulness of AnGeLi by contributing additional published gene lists via its data submission feature. Such community submissions will enhance the visibility and citations of the papers reporting the submitted lists, and will help to unleash the full power of genomic data sets.

## Author Contributions

DB, FS, and JB conceived the study. FS developed the prototype of AnGeLi and wrote the core Perl modules. DB extended its functionality, improved Perl-cgi scripts, and wrote all the R scripts needed for creation and update of AnGeLi’s database. DB also integrated multiple annotation resources, curated the majority of data features, and configured the web server. MO wrote the recursive R function needed for traversing the ontology graphs. SK and SD improved the user interface. GS helped fine-tuning AnGeLi’s performance. VW helped in designing the tool and improving its functionality. VP wrote the scripts for retrieval of pairwise data type and amino acid composition. DB and JB wrote the manuscript.

## Conflict of Interest Statement

The authors declare that the research was conducted in the absence of any commercial or financial relationships that could be construed as a potential conflict of interest.

## Abbreviations

AnGeLi: Analysis of Gene Lists
BioGRID: Biological General Repository for Interaction Datasets
EMBOSS: European Molecular Biology Open Software Suite
FDR: False Discovery Rate
FYPO: Fission Yeast Phenotype Ontology
GO: Gene Ontology
GSEA: Gene Set Enrichment Analysis
Pfam: Protein Families
